# Perception and Motion in Real and Virtual Environments: A Narrative Review of Autism Spectrum Disorders

**DOI:** 10.3389/fpsyg.2021.708229

**Published:** 2021-07-12

**Authors:** Irene Valori, Phoebe E. McKenna-Plumley, Rena Bayramova, Teresa Farroni

**Affiliations:** ^1^Department of Developmental Psychology and Socialization, University of Padua, Padua, Italy; ^2^School of Psychology, Queen’s University Belfast, Belfast, United Kingdom; ^3^Department of General Psychology, University of Padua, Padua, Italy

**Keywords:** sensorimotor development, virtual reality, IVR, HMD, autism, ASD, perception, motion

## Abstract

Atypical sensorimotor developmental trajectories greatly contribute to the profound heterogeneity that characterizes Autism Spectrum Disorders (ASD). Individuals with ASD manifest deviations in sensorimotor processing with early markers in the use of sensory information coming from both the external world and the body, as well as motor difficulties. The cascading effect of these impairments on the later development of higher-order abilities (e.g., executive functions and social communication) underlines the need for interventions that focus on the remediation of sensorimotor integration skills. One of the promising technologies for such stimulation is Immersive Virtual Reality (IVR). In particular, head-mounted displays (HMDs) have unique features that fully immerse the user in virtual realities which disintegrate and otherwise manipulate multimodal information. The contribution of each individual sensory input and of multisensory integration to perception and motion can be evaluated and addressed according to a user’s clinical needs. HMDs can therefore be used to create virtual environments aimed at improving people’s sensorimotor functioning, with strong potential for individualization for users. Here we provide a narrative review of the sensorimotor atypicalities evidenced by children and adults with ASD, alongside some specific relevant features of IVR technology. We discuss how individuals with ASD may interact differently with IVR versus real environments on the basis of their specific atypical sensorimotor profiles and describe the unique potential of HMD-delivered immersive virtual environments to this end.

## Introduction: Multisensory Development

Embracing a neuroconstructivist approach, we can conceive of the development of an individual as a continuous and dynamic process of interaction among genetic constraints and environmental landscapes through the plasticity of the brain (Karmiloff-Smith, 2009). Experience guides an individual’s developmental trajectory in a probabilistic epigenesis and shapes the brain through progressive specialization for elaborating certain types of stimuli (Johnson, 2001). Our senses are the entry gates by which a given stimulation can be perceived, elaborated, and qualified as experience that then constructs the individual phenotype. Numerous sensory inputs present in our environment at any given time have to be bounded by the principles of spatial, temporal, and semantic congruence. This multisensory processing specializes with age so that the developmental stage establishes which information has to be elaborated and how and when different inputs are combined (Bremner et al., 2012). Furthermore, information coming from different modalities contributes to the formation of an aggregate percept to a different extent depending on which modality is the most precise and appropriate to the given context, goal, and task. This is also fundamental in motor development, which requires the combination of exteroceptive (i.e., vision), proprioceptive, and vestibular cues, and vestibular cues, on the basis of sensory-sensory and sensory-motor contingencies, namely the co-occurrence of multimodal stimuli in the same spatio-temporal window (Murray et al., 2016), and the correspondence between sensory feedback and motor output (Baldassarre et al., 2018).

To deal with the uncertainty of multimodal combination and integration (e.g., in case of discordant, ambiguous, or missing information), the mind has to base its reasoning on prior experience and decide which is the most plausible interpretation of several possibilities (Ernst and Bülthoff, 2004). The use of prior information in perception has been subject to extensive investigation and also modeled within the Bayesian framework (Pellicano and Burr, 2012). In an attempt to describe the processes underlying the derivation of the most probable interpretations of the environment, Pellicano and Burr (2012) suggest formalizing sensory atypicalities in Autism Spectrum Disorders (ASD) using this mathematical framework. In particular, while neurotypical toddlers show limited multisensory integration, which develops up to adolescence and results in a sort of mandatory integration of either congruent or incongruent cues, adolescents with ASD present a more selective multisensory integration only for congruent stimuli (Bedford et al., 2016). This has been interpreted as enhanced perceptual functioning, whereby sensory inputs are weighted more than prior or contextual knowledge in building up perception (Palmer et al., 2017).

In sum, rather than a precise detector of reality, the human mind is a simulation system that utilizes prior knowledge to build expectations of the world. It goes without saying that mistakes are honored guests at this guessing game. As a consequence, the mind is the victim of many errors, crashes, and bugs in its predictive coding: false memories, illusions, attentional blindness, cognitive biases, heuristics, and so on (Buonomano, 2011).

## Immersive Virtual Reality

**FIGURE 1:**
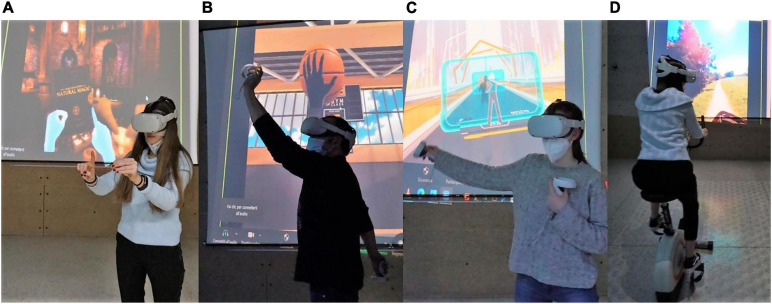
(from left to right): Users wearing HMDs and interacting with commercially available applications through their actual hands **(A)**, controllers in their hands’ place and their virtual hand-like representation **(B)**, their whole body as represented by a virtual full-body shadow **(C)** or external movement platforms (i.e., bicycle) **(D)**.

As the mind is a simulation system that filters reality with the precise goal of coming up with a coherent interpretation of the world, we have considerable chances of hacking the process and making people perceive, feel, and believe something unreal. Immersive Virtual Reality (IVR) technologies have been extensively studied in terms of their potential for manipulating the boundaries of the mind. Notably, “virtual worlds are constructed by the senses and only really exist in the mind of users. VR is a medium for the extension of body and mind” (Biocca and Delaney, 1995, p. 58). Indeed, the body and mind can be extended through IVR: the technology has been used to produce the Rubber-Hand Illusion (RHI) (Yuan and Steed, 2010), to make users feel that they are in someone else’s body (Slater et al., 2010), and even to change feelings, behaviors, and attitudes (Maister et al., 2015; Bergström et al., 2016). When talking about VR, one highly important point of discussion is the degree of objective immersion and subjective sense of presence that the device induces. One of the most immersive systems available which is relatively affordable is the wearable head-mounted display (HMD), which excludes the visual and auditory real world and engages the user in a virtual simulation made of 360° stereoscopic environments and binaural spatialized audio. The attractiveness of HMDs is also due to the fact that they offer the possibility for full body movements and navigation (Figure 1).

For many years, researchers stated that individuals’ performance within IVR mirrored their functioning in real settings (Slater et al., 2009; Iachini et al., 2016; Maffei et al., 2016). On the other hand, recent studies are highlighting that, even though this technology is continually and exponentially improving, users’ experiences with HMDs do not perfectly resemble the sensorimotor features of reality. For instance, the way HMDs provide visual information may require a different type of sensory processing, thus impacting the perception-action link. Scarce cues to depth and almost absent haptic feedback may create visual environments that primarily activate the ventral visual path for perception and recognition of stimuli, thus impairing the dorsal path that is specialized for the visual control of actions (Harris et al., 2019). The issue of compromised visual processing or higher load on the visual networks than in real visual environments to achieve aspects of visual cognition like depth perception is currently an active area of empirical testing and applied research (Fulvio et al., 2020). At the neural level of multisensory integration, a recent electroencephalography (EEG) study on the RHI pointed out that the illusion might induce different oscillatory underpinnings when achieved through real stimuli or IVR. The authors suggest that the integration of spatially congruent visuo-tactile information requires additional cognitive control in IVR compared to real settings, as if there were some sort of unresolved mismatch between the two modalities (Kanayama et al., 2021). Evidence from functional magnetic resonance imaging (fMRI) also shows that brain activity related to spatial processing (assessed through participants’ judgments of whether 3D objects were centered, shifted to the left or to the right) is different in IVR as compared to known brain activations in reality (Beck et al., 2010). Ultimately, there is mounting awareness of the importance of multimodality in designing and employing virtual environments for specific purposes, as well as in assessing the ecological validity of IVR (Xu et al., 2021).

Additionally, it has been suggested that self-motion can be altered when people interact with IVR through HMDs (Willemsen et al., 2004; Nilsson et al., 2018). Indeed, tasks requiring actual locomotion and rapid head movement increase the occurrence of well-known after-effects such as motion sickness, mainly due to the tracking latency that causes a temporal lag between the user’s movement and the consequent change in the optic flow (Allison et al., 2001). A main departure from self-motion and navigation in reality is arguably the lack of our own body in the visual field, which affects the three components of embodiment: the sense of being where the (virtual) body is, the sense of agency and control over the (virtual) body and environment, and the sense of ownership over the (virtual) body (Kilteni et al., 2012). Studying the role of including a virtual body (VB) representation in IVR has long been considered an important issue in the available literature (Slater and Usoh, 1993). With advanced technologies, HMDs can make the user see themselves from a first-person view and can include the presence of a graphical representation of the body at different levels of realism: a geometric indicator of the user’s position in space, a representation of the user’s hands, the shadow of the user, or even a full body self-avatar (Pan and Steed, 2017). In this way, HMDs can play an important role in presence, which is greatly discussed in the literature on IVR. Presence refers to the degree to which a user feels present and located within the virtual reality – it might be indicated by the “looming effect,” where users react in the real world to actions that are occurring in the virtual reality (George et al., 2018). Various factors affect presence, such as the user having a virtual body (Slater and Usoh, 1994), using real-world navigation techniques like walking (Slater et al., 1995), and the ability to generate vivid mental images (Iachini et al., 2019). The existing evidence seems to indicate that enhancing visual realism in VB representations could be important to induce realistic responses, possibly thanks to the enhanced sense of presence they create (Slater et al., 2009).

However, one recent study suggests that visuo-proprioceptive congruency could be more central than visual fidelity (Zopf et al., 2018). The authors manipulate the visual realism of the VB by showing the participant a virtual hand or a sphere. They also manipulate visuo-proprioceptive congruency: the VB can move consistently or inconsistently with the user’s real body movements. Both factors are implicated in the sense of presence, but only visuo-proprioceptive congruency is crucial for the sense of agency. This leads to the ability to manipulate visual realism while maintaining a high sense of “acting there”. In this regard, VB visual fidelity has been intentionally altered by some researchers to affect the user’s perceptions, attitudes, or behaviors. The body swapping method allows researchers to expose the user to a different physical self. The users see a different human body from the first-person perspective, and experience a successful illusion of ownership that has been consistently reported via self-report and physiological measures (Petkova and Ehrsson, 2008). Petkova and Ehrsson’s (2008) study provides additional evidence of the need for perceptual congruence. The authors used visuo-tactile stimulation that induced the illusion of ownership only in the case of synchronous stimulation. Asynchronous visuo-tactile stimulation does not induce such an illusion. After this body swapping exposure, users appeared to even show changes to the memory of their bodies, with interesting clinical applications, for example for patients with eating and weight disorders (Serino et al., 2016).

In sum, recent evidence points out that VR differs from real environments for both low-level sensory processes and higher-order cognitive aspects. Indeed, while sensory information in VR differs from reality in terms of higher perceptual uncertainty, even in perceptually “perfect” virtual environments individuals’ prior knowledge of acting in a virtual rather than real world influences their expectations of action consequences, thus affecting behaviors (Giesel et al., 2020).

## Autism Spectrum Disorders

Sensory atypicalities are early risk factors which confer cascading effects on child development, potentially marking the onset of neurodevelopmental difficulties and disorders (Hill et al., 2012). ASD are characterized by atypical sensory processing that may have subsequent effects on the later development of higher-order cognitive and social abilities (Baum et al., 2015). Currently, ASD is diagnosed based on persistent and pervasive deficits in social communication and social interaction, as well as restricted and repetitive patterns of behaviors, interests, or activities. The latter category of symptoms consists of repetitive motor movements, use of objects, or speech; insistence on sameness, routines, or rituals in verbal or non-verbal behaviors; restricted, repetitive patterns of behaviors or intensely focused interests and preoccupations, and hyper/hypo-reactivity to sensation (American Psychiatric Association (APA), 2013). Children with ASD present different types of sensory symptoms, such as hyper- or hyposensitivity, unique patterns of response to sensory stimuli, sensory seeking (Baranek et al., 2006), and reduced discrimination between novel and repetitive stimuli investigated by habituation paradigms (Vivanti et al., 2018). This type of heterogeneity in sensory responsiveness implies the existence of subtypes within the autism spectrum (Schoen et al., 2008), with each potentially having different cascading effects on other areas of cognitive and social functioning (Raymaekers et al., 2004; Schultz, 2005). Based on the direction of these interconnections between sensory, cognitive, and social events, specific sensory training programs could be designed through careful identification of which level of stimulation is appropriate for different individuals in each sensory modality.

People with ASD present unique processing of unimodal stimuli, such as higher temporal binding of visual cues from two years of age (Freschl et al., 2020), reduced sound tolerance (Williams et al., 2021), atypical brain responses to both affective and non-affective touch (Kaiser et al., 2016), olfactory dysfunctions (Crow et al., 2020), and peculiar taste reactivity (Avery et al., 2018). Together with atypicalities in the individual sensory channels, people with ASD show broad differences at the multisensory level (Hill et al., 2012; Baum et al., 2015). Researchers have reported reduced multisensory facilitation and higher reliance on unimodal processing (Collignon et al., 2013), an extended (hence less precise and specialized) multisensory temporal binding window (Foss-Feig et al., 2010), reduced integration of audio-visual cues (Feldman et al., 2018), atypical integration of interoceptive and exteroceptive stimuli such as reduced cardio-visual temporal acuity (Noel et al., 2018), and delayed or reduced effects of visuo-tactile stimulation on proprioception during the RHI (Cascio et al., 2012; Greenfield et al., 2015). Therefore, there is a widespread interest in interventions that focus on the training of sensory processing in ASD. Although it has been suggested that multisensory function may be malleable with treatment, there is a relative lack of evidence that treatment improves this functioning in people with ASD (Cascio et al., 2016). The existing body of research does not support the use of those therapies that simply provide additional possibilities to obtain visual, tactile, vestibular, or proprioceptive sensory stimulation, and challenge motor control to promote adaptive behaviors (for example, the so-called Sensory Integration Therapy; for a review, see Lang et al., 2012). It suggests that more elaborate tasks should be designed in order to ensure concentration on certain abilities and control for progress.

Another aspect to consider when targeting a specific set of skills is thorough examination of areas where the individual functions within the normal range or even more accurately. For example, one may focus on training various visual processing mechanisms that lead to an efficient use of visual landmarks for successful motor output based on studies showing that impairments in vision are associated with lower sensorimotor performance. This has been indicated in a number of studies where the contributions of vision and proprioception were studied in unisensory and multisensory conditions in spatial cognition tasks and in which participants with ASD have been found to rely more on proprioception (Haswell et al., 2009; Marko et al., 2015) from childhood (Izawa et al., 2012) to adulthood (Morris et al., 2015). Although it may seem that such individuals with ASD need remediation only in the visual domain, the neuroconstructivist approach would include rehabilitation of proprioception as well since this efficient (or superior) proprioceptive functioning in ASD may originate from an atypical focus on certain sensory features that leads to the deterioration of other skills. This approach is at the core of the neuroconstructivist school of thought which emphasizes the influence of the developmental trajectory of one sensory modality/skill on the other (Karmiloff-Smith, 2009).

From an embodied cognition perspective, multisensory development goes hand-in-hand with motor development, in a perception-action cycle that allows the individual to learn and explore both the self and the external world (Kiefer and Trumpp, 2012). In concert with multisensory functioning, physical and motor development set out age-specific constraints and sensitive periods for the possibility of learning certain skills. By exploring and acting as an agent within the world, children develop mechanisms that enable optimal integration between sensory input and motor output. Motor development is not a trivial acquisition of milestones, but a complex self-organization challenge to integrate the mechanical part of the body with perceptions, thoughts, emotions, and their physiological underpinnings (Thelen, 1989). From infancy, babies at high risk for later diagnosis of ASD manifest delayed and qualitatively different motor development. This is a pervasive and consistent phenomenon, as highlighted by a recent meta-analysis (West, 2019). Later in life, children with ASD show a variety of motor impairments in the domains of praxis and fine and gross motor skills (Kaur et al., 2018). Toddlers with ASD also seem to present asymmetrical gait (Esposito et al., 2011), and impaired postural stability has been found up to adolescence and adulthood in one-leg standing (Travers et al., 2013). The postural deficit seems to be quite established in literature, according to a systematic review and meta-analysis of 19 studies (Lim et al., 2017). Given the unbreakable link between the development of sensorimotor processes and higher-order operations, options for sensorimotor interventions need to be explored.

## IVR and Sensorimotor Functioning in ASD

In recent years, there has been mounting interest in the investigation of the potential that digital and multimedia technologies might have for sensorimotor stimulation of people with ASD. Encouraging indications come from projects such as the European-funded MultiSensory Environment Design for an Interface between Autistic and Typical Expressiveness (Pares et al., 2005), the Magic Room: A Smart Space for Children with Neurodevelopmental Disorder (Garzotto et al., 2019), and the Lands of Fog (Crowell et al., 2020). These teams realized mixed realities and multimedia interactive environments that foster children’s sense of agency, provide sensorimotor stimulation, and are feasible even with low-functioning individuals with ASD. However, they did not utilize fully immersive VR, and the extant literature is far from exhaustive, as few studies have employed immersive tools rather than computers and screens and most studies have small samples, no control group, and primarily focus on social, daily-life, and safety skills (Lorenzo et al., 2019). Some authors have made an attempt to include sensorimotor aspects among the design considerations of VR applications for individuals with ASD, thus suggesting limited use of sudden loud sounds, sound control for the user, and use of sharp visuals that include colors, shapes, and movement of stimuli (Bozgeyikli et al., 2018). It has been suggested that HMDs, rather than monitors, enhance spatial presence and are preferred by children with ASD (Malihi et al., 2020b). However, this sense of presence might be modulated by individual factors such as IQ and anxiety, whereby a higher IQ seems related to greater sense of presence and engagement only in children with low levels of anxiety (Malihi et al., 2020a). This underlines the importance of attending to inter-individual differences when considering interaction with IVR.

A few studies have recently explored whether HMDs could stimulate different sensory systems in people with sensory conditions. Some researchers have received positive feedback from occupational therapists who used customizable HMD-games for children with sensory processing disorders. Different games were designed to stimulate several sensory systems through manipulation of visual properties (complexity of the environment, object color and size), audio volume and effects, and vestibular input (e.g., speed of participant’s roller-coaster cart) (Rossi et al., 2019). However, the effects on participants’ sensorimotor functioning were not directly measured. HMDs have also been employed to deliver contextual sensory integration therapy and train adults with vestibular disorders in sitting, standing, turning, or stepping within different scenarios (i.e., city, park, airport). Therapists used the HMD together with their typical rehabilitation methods, and participants compiled self-report questionnaires that indicated improvements in vertigo and balance (Lubetzky et al., 2019). No direct measures of patients’ performance were included, and the absence of a control group that did not undergo the HMD training prevents us from attributing these benefits specifically to the IVR. In the auditory domain, in order to address auditory hypersensitivity, 6 adolescents with ASD have been exposed to IVR games whereby they encountered three-dimensional, spatialized sounds that they found anxiety-provoking (according to self and parents’ reports). Participants joined four weekly 30-min desensitization sessions, whereby each stimulus could be delivered a maximum of 20 times and was gradually moved closer to the participant, thus reducing their perceived anxiety and increasing the interaction time towards the target stimuli (Johnston et al., 2020). Despite these preliminary works offering encouraging suggestions, further investigation is needed to elucidate the sensorimotor potential of IVR training, which would be particularly compelling for ASD, and their acceptability to users (Valori et al., 2020a).

Together with the interest in addressing sensorimotor processing in people with ASD, only in the past decade have researchers begun to investigate how individuals with ASD perceive through HMDs. For instance, children and adolescents with ASD seem to benefit from binaural spatialized audio when exploring virtual environments with HMDs (Johnston et al., 2019). On the other hand, some evidence suggests that adults with ASD compared to controls are less susceptible to the full body illusion in IVR, not demonstrating the embodiment in a VB (Mul et al., 2019). The lack of embodiment has been found to be associated with severity of ASD traits and reduced peripersonal space, which is the space immediately around our body in which actions are possible (for a review on peripersonal space, see Holmes and Spence, 2004). Indeed, given the importance of visuo-proprioceptive congruency to induce a sense of agency in IVR (Zopf et al., 2018), atypical visuo-proprioceptive integration in ASD (Oldehinkel et al., 2019) might underlie the limited sensitivity to the VB illusion. The authors suggest that an atypical body awareness might be related to multisensory integration difficulties, with potential adverse effects on social abilities (Mul et al., 2019). For instance, higher reliance on body-based interoceptive signals impairs sensitivity to body illusions due to a limited use of external information, which is fundamental to interact with people and objects around us (Schauder et al., 2015). The balance between processing and perceiving what is happening *inside* or *outside* the self is at the heart of social cognition processes of understanding both the similarity and distinction between the self and the others (Palmer and Tsakiris, 2018).

Ultimately, the pending question here is to what extent these results are related to aspects or features of ASD and/or HMDs. To shed light on this topic, the performance of individuals with ASD has to be compared between equivalent real and IVR environments. To the best of our knowledge, this has only been investigated with respect to social behaviors by Simões et al. (2020) and in terms of sensorimotor processing by our research team (Valori et al., 2020b). Children with ASD appeared to feel comfortable with the same interpersonal distance between them and either a real or virtual character in an HMD-delivered IVR designed to be a faithful reproduction of the real one (Simões et al., 2020). In a recent pilot experiment, we studied how children and adults with ASD move and perceive their own movements with different sensory information available in IVR versus real environments (Valori et al., 2020a). Seven participants with ASD were rotated to a certain degree while sitting on a swivel chair, and then asked to actively rotate back to the starting position, thus encoding and reproducing the exact self-motion. The task was performed in two environments (Reality and IVR) for each of three sensory conditions (Only Proprioception, Only Vision, Vision + Proprioception). In our sample, those with higher accuracy when visual cues were available performed better in reality. On the other hand, participants who were facilitated by moving when only proprioception was reliable (e.g., in blindfolded conditions) showed higher self-motion accuracy in IVR compared to reality. The latter cases of facilitation for moving without vision appeared to be atypical compared to the strong reliance on vision that characterized participants with typical development across all age groups in a previous stage of our study (Valori et al., 2020b). Our exploratory findings highlight that inter-individual variability in sensorimotor functioning might have a relevant impact on the possibility for people with ASD to be facilitated by perceiving, moving, and therefore learning in IVR.

## Discussion

The unique way in which HMDs provide multisensory cues and require specific prior knowledge about the implications of acting in a virtual rather than real world might help to explain why this technology could be specifically useful for those individuals with impairments in sensory processing. To the best of our knowledge, nothing is known about how these aspects could affect the distinctive way in which individuals with ASD weight sensory inputs and prior or contextual knowledge in building up perception (Palmer et al., 2017). In addition, while virtual environments have been suggested to reduce the engagement of the dorsal visual stream in favor of the ventral one (Harris et al., 2019), further research is needed to understand this effect on the atypical visual functioning reported in ASD (Grinter et al., 2010). Furthermore, technical aspects such as the display lag in tracking head position in space (Allison et al., 2001) might allow HMDs to enlarge the temporal window between stimuli, thus facilitating multisensory integration for people with ASD, who manifest an enlarged multisensory temporal binding window (TBW). Indeed, while the width of the TBW can be narrowed through temporal discrimination training (Zhou et al., 2018), this “remediation approach” leaves open the question of whether we could rather embrace a neuroconstructivist view and also provide people with environments suited to their individual TBW. From this perspective, we could speculate that an enlarged inter-stimuli delay might reduce the “multisensory crowding” associated with enlarged TBWs, resulting in sensory and learning facilitation. Although this has yet to be investigated, our studies suggest that IVR and HMDs disrupt proprioception in neurotypical groups (Valori et al., 2020b) and can either improve or worsen sensorimotor performance in individuals with ASD depending on the way they utilize visual and proprioceptive information, which differs across individuals (Valori et al., 2020a).

It is worth mentioning that the present paper primarily reviews literature that employed HMDs, which are the most widespread, cost-effective, and easy to use immersive technology. However, there are other systems which are generally defined as immersive, such as the Cave Automatic Virtual Environment (CAVE), whereby up to six synchronized projection screens create a room of multimedia contents and visuals that can be perceived in three dimensions through stereoscopic glasses (Cruz-Neira et al., 1992). It is well known that the two devices differ in the way they provide proprioceptive input (i.e., giving or not giving the users a weight on their head) and visual stimulation, with different field of view, eyes-screen, and participants-objects-screen distances that might reasonably affect eyes’ accommodation, users’ distance and depth perception, and even the sense of immersion and presence (Naceri et al., 2010; Combe et al., 2021). Moreover, despite being immersive and interactive, the CAVE does not completely separate users from the physical world, thus allowing them to perceive and see their own bodies almost as usual. To our knowledge, there is not much evidence in the extant literature to shed light on the potential discrepancies and similarities in the way individuals with ASD perceive and move in HMDs or other immersive environments. Although no direct comparisons between HMDs and CAVEs have been explored with this population, some authors investigated the combination of vision and vestibular senses in participants with ASD interacting with a CAVE system. In a sample from Greffou et al. (2012), 12- to 15-year-old adolescents with ASD, compared to a neurotypical control group, showed less postural reactivity to a visual stimulation aimed at inducing vestibular instability through high frequency oscillations of a virtual tunnel presented inside a CAVE. This difference between experimental and control groups was not detected in older participants (16–33 years) (Greffou et al., 2012). These results appear consistent with those collected in real environments, whereby adults with ASD showed a reduced contribution of vision in adapting to an induced postural illusion (Morris et al., 2015). Further research is needed to disentangle the contributions of different technologies (e.g., CAVE vs. HMD vs. multimedia environments) to sensorimotor performance in IVR environments, including a consideration of which technologies are most acceptable to users. However, thus far, it appears that HMDs may offer a promising and cost-effective option.

In conclusion, IVR technology is continually improving and has shown the ability to alter perception, behavior, and attitude. It can also be used to study sensorimotor processing in terms of both unisensory and multisensory inputs. Given the atypical sensorimotor functioning evidenced in individuals with ASD and previous successful uses of HMD-delivered IVR in this population, it appears that IVR has some specific potential for both studying and training these abilities in people with ASD. However, there is not one clear pattern of processing used by individuals with ASD, and researchers and clinicians aiming at the design and implementation of IVR training should be aware of the individual processing styles of the target users in order to effectively tap their needs, strengths, and weaknesses.

## Ethics Statement

Written informed consent was obtained from the individuals for the publication of any potentially identifiable images or data included in this article.

## Author Contributions

IV, PM-P, RB, and TF: conceptualization, writing the original draft, review, and editing. All authors contributed to the article and approved the submitted version.

## Conflict of Interest

The authors declare that the research was conducted in the absence of any commercial or financial relationships that could be construed as a potential conflict of interest.
